# Stereotactic two-needle irreversible electroporation of liver tumors near critical structures: a proof-of-concept study

**DOI:** 10.1186/s41747-025-00674-y

**Published:** 2026-02-23

**Authors:** Liang Zhang, Vinzenz Mayr, Lukas Luerken, Quirin Strotzer, Moritz Brandenstein, Laura Kupke, Anthony Ngu, Christian Stroszczynski, Ingo Einspieler

**Affiliations:** Department of Radiology, University Hospital Regensburg, Regensburg, Germany

**Keywords:** Ablation techniques, Carcinoma (hepatocellular), Colorectal neoplasms, Electroporation, Irreversible electroporation

## Abstract

**Objective:**

Irreversible electroporation (IRE) is a non-thermal ablation technique suitable for tumors near critical structures, but its widespread use is limited by technical complexity and the need for multiple electrodes. This study aimed to evaluate the feasibility, safety, and efficacy of a stereotactic percutaneous two-needle IRE approach for small liver tumors in anatomically challenging locations.

**Materials and methods:**

In this retrospective study, 17 consecutive patients with 18 primary or secondary liver tumors (≤ 2.0 cm) adjacent to critical anatomical structures underwent CT-navigated stereotactic two-needle IRE between December 2021 and May 2025. Ablation was performed with a high-dose protocol (2 × 90 pulses, 90 µs, > 20 A). Primary endpoints were primary technique efficacy (PTE) and local tumor progression (LTP); secondary endpoints included complications. Needle placement was assessed through geometric analysis.

**Results:**

PTE was obtained in 17/18 tumors (94.4%, 95% confidence interval (CI): 72.7–99.9%). At a median follow-up of 12.4 months, LTP occurred in 1/18 tumors (5.6%, 95% CI: 0.1–27.3%). No complications or procedure-related mortality were observed. Geometric analysis showed high accuracy of stereotactic needle placement, while treatment failure was associated with suboptimal geometry.

**Conclusion:**

Stereotactic percutaneous two-needle IRE seems to be technically feasible with a favorable safety profile for small liver tumors in anatomically challenging locations and may offer a simplified alternative to multielectrode approaches. However, given the small, retrospective single-center design, these findings are preliminary and require prospective multicenter validation to establish oncologic effectiveness and generalizability.

**Relevance statement:**

Stereotactic two-needle irreversible electroporation offered a simplified, safe, and effective alternative to multielectrode ablation, potentially broadening treatment options for liver tumors near critical structures and improving accessibility, reproducibility, and outcomes in interventional oncology.

**Key Points:**

- First systematic clinical evaluation of stereotactic two-needle irreversible electroporation (IRE) for liver tumors.
- Two-needle configuration with high-dose protocol simplifies IRE compared with standard multielectrode approaches.
- This proof-of-concept study demonstrates high efficacy and absence of complications in small liver tumors near critical structures.
- Two-needle IRE may broaden clinical applicability in anatomically challenging locations.

**Graphical Abstract:**

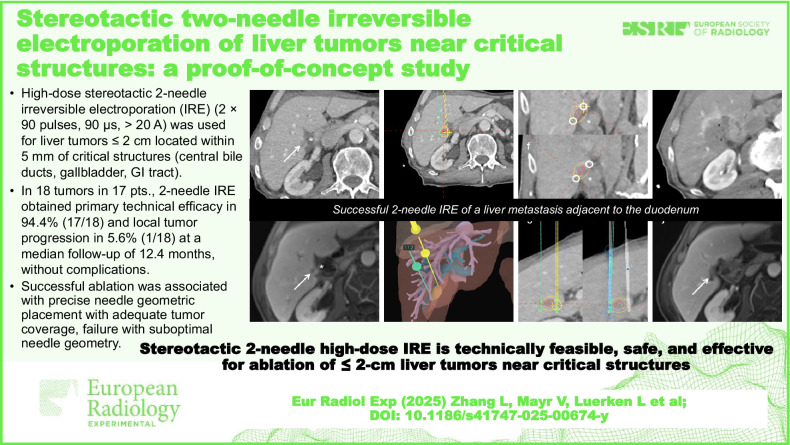

## Background

Image-guided tumor ablation has become an integral component in the treatment of primary and secondary liver malignancies [1–5]. Among the most established modalities are thermal ablation techniques such as radiofrequency ablation (RFA) and microwave ablation (MWA). While effective, these techniques pose a risk of collateral damage when tumors are located near critical structures such as the central bile ducts, gallbladder, or gastrointestinal tract [6–10]. These proximity-related risks are acknowledged in contemporary guidelines, which prioritize thermal ablation [1–5].

Against this backdrop, non-thermal ablation approaches are being explored as potential alternatives in anatomically challenging cases, notably irreversible electroporation (IRE), which induces cell death by creating permanent nanopores in the cell membrane using high-voltage, short-duration electric pulses, while preserving the extracellular matrix and minimizing collateral damage [11–16]. This unique mechanism makes IRE particularly advantageous for tumors near critical structures, where alternative treatments are limited [16–22]. However, the widespread use of IRE is limited by procedural complexity. Achieving a sufficient and uniform electric field in conventional IRE typically requires the parallel placement of three or more electrodes, a demanding task where even small deviations in needle geometry can significantly compromise treatment efficacy [23–26]. This multineedle requirement often prolongs planning and anesthesia time, increases workflow complexity, and may elevate complication risk, particularly in anatomically constrained regions.

Stereotactic and robotic navigation systems can help overcome this challenge by enabling meticulous preoperative planning and real-time image guidance to improve placement accuracy [27–29]. However, even with stereotactic assistance, the complexity of multineedle IRE remains a significant barrier to widespread use. A complementary strategy to simplify the procedure is to reduce the number of electrodes required for effective ablation. A two-needle IRE approach could significantly reduce technical complexity, procedural time, and overall cost, while potentially making the technique more accessible to a broader range of patients and clinical settings.

However, performing IRE with only two needles may introduce inherent technical limitations, including a smaller ablation zone and potential difficulties in achieving uniform electric field distribution [30]. It is conceivable that these limitations could be mitigated by a combination of strategies, including (1) precise stereotactic planning with parallel needle placement to optimize electric field geometry, (2) careful adjustment of ablation parameters, such as increasing electrical field strength and amount of pulses, and (3) restricting use to small tumors where complete ablation is more likely. Under these conditions, a two-needle IRE technique could offer a safe and effective alternative for selected patients.

While most IRE studies have focused on multielectrode configurations [28, 31–37], and some animal studies have examined two-needle configurations in preclinical models [38, 39], no clinical study to date has systematically evaluated a dedicated two-needle configuration for human liver tumors.

We conducted a retrospective, proof-of-concept, exploratory study to evaluate a stereotactic percutaneous two-needle IRE technique for small primary or secondary liver tumors near anatomically critical structures. Unlike conventional multielectrode IRE, which can prolong procedure time, increase procedural complexity, and raise complication risk in anatomically constrained regions, we examined a dedicated two-electrode configuration. The primary aim was to assess the technical feasibility, safety, and preliminary efficacy of this simplified method. Additionally, we performed a detailed geometric analysis to evaluate whether optimal needle placement can be consistently achieved with this configuration.

## Materials and methods

### Patients and study design

This study was approved by the institutional ethics committee of the University of Regensburg (approval no. 25-4291-104, approved on 06 August 2025) and conducted in accordance with the Declaration of Helsinki.

This single-center, retrospective study included all patients who underwent stereotactic, percutaneous two-needle IRE for primary or secondary liver tumors between December 1, 2021, and May 31, 2025, identified from institutional procedure logs and electronic medical records. Case accrual was consecutive; all patients who received the two-needle IRE procedure within the study window were analyzed.

Before scheduling IRE, all candidates were screened with a standard preprocedural checklist: (1) tumor criteria: single lesion or ≤ 2 lesions, each ≤ 2.0 cm and ≤ 5 mm from critical structures (central bile ducts, gallbladder, gastrointestinal tract); (2) no prior local liver therapy to the target lesion; (3) Child-Pugh score < 10; and (4) no general contraindications to IRE (*e.g*., intolerance to general anesthesia, severe arrhythmia, pacemaker dependence or other at-risk implanted devices, uncorrectable coagulopathy, active systemic infection, pregnancy).

All cases were reviewed by the institutional multidisciplinary tumor board (MTB), which confirmed the indication and modality by consensus. Thermal ablation (RFA/MWA) was preferred for non-resectable lesions when a safe margin could be achieved without jeopardizing critical structures. When adequate thermal margins were unlikely, typically for lesions within ≤ 5 mm of critical structures, the MTB indicated non-thermal ablation with IRE. For lesions ≤ 2.0 cm with an MTB indication for IRE, our predefined institutional technique was stereotactic two-needle IRE to reduce procedural complexity; lesions > 2.0 cm with an IRE indication were treated with conventional multielectrode IRE.

Importantly, the initial two-needle IRE did not restrict access to our institution’s established standard non-thermal modalities, conventional multielectrode IRE and electrochemotherapy (ECT). Non-PTE at first follow-up or LTP during surveillance triggered MTB re-review and retreatment with multielectrode IRE or, when appropriate, ECT.

### Stereotactic IRE

All IRE procedures were performed in an interventional computed tomography (CT) suite (Somatom Definition Edge, Siemens Healthineers). Procedures were conducted under general anesthesia with endotracheal intubation and complete neuromuscular blockade. To ensure a stable stereotactic reference, respiratory motion was controlled using standardized end-expiratory apneic pauses: the anesthesiologist paused ventilation at end-expiration for planning CT, needle advancement, and verification scans, then resumed between steps. Patients were positioned supine or slightly elevated in the right lateral position on a vacuum fixation mattress. After sterile preparation, radiopaque skin fiducials were applied. A contrast-enhanced planning CT scan (arterial and portal-venous phases) was acquired during temporary apnea following intravenous administration of 100 mL of non-ionic iodinated contrast (Accupaque™ 350; iohexol 350 mg I/mL; GE Healthcare Buchler GmbH & Co. KG). The image dataset was transferred to the CAS-One IR stereotactic navigation platform (CAScination AG), positioned adjacent to the CT scanner.

Using CAS-One IR software (CAScination AG), the tumor was segmented, and trajectories for the two IRE needles were planned. If the tumor was not sufficiently visible on the planning CT, image fusion with prior contrast-enhanced CT or magnetic resonance imaging (MRI) was performed. Needle trajectories were planned to be as parallel as possible, targeting the tumor margin with an active tip length of 2 cm. When feasible, needle tips were positioned slightly beyond the tumor boundary to ensure complete coverage while avoiding direct contact with critical structures.

IRE procedures were carried out using the Dophi™ N3000 Electroporation System (Surgnova Healthcare Technologies). Under stereotactic guidance, two 19-gauge electrodes were percutaneously inserted. Needle placement was confirmed using non-contrast CT and adjusted as needed to ensure optimal alignment with the planned trajectory.

After confirming needle placement, a test sequence of 10–20 pulses (pulse length 90 µs) was delivered with the electric field strength adjusted to achieve a target current > 20 A, aiming for a therapeutic range of 21–30 A, within the system’s maximum output of 3,000 V. The therapeutic protocol consisted of two consecutive cycles of 90 pulses each (90 µs, up to 3,000 V). All pulses were synchronized to the absolute refractory period of the cardiac cycle using electrocardiogram gating.

**Fig. 1:**
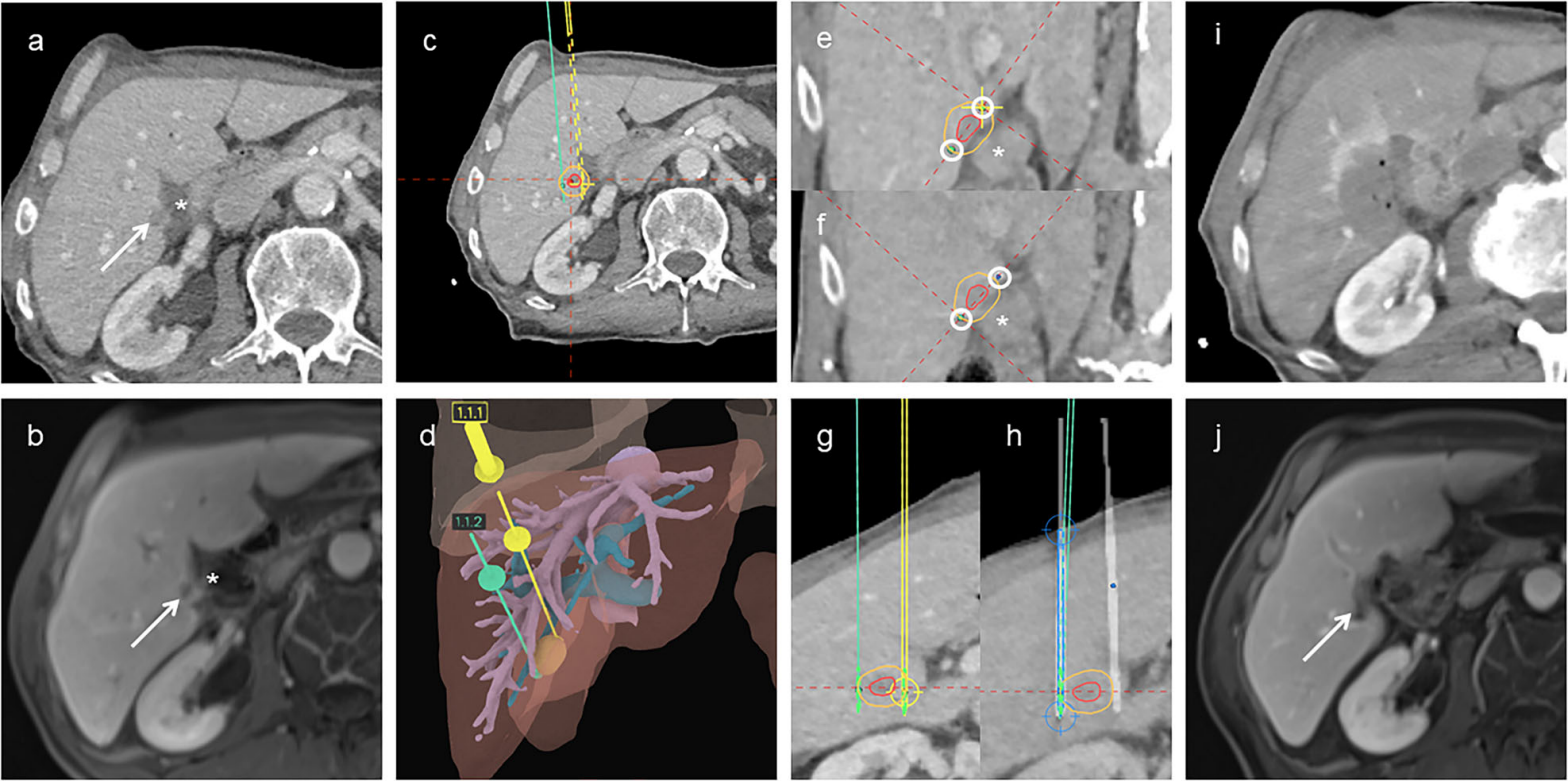
Stereotactic two-needle irreversible electroporation in a patient with a pancreatic metastasis near the duodenum. **a**, **b** Axial contrast-enhanced CT and MRI showing the tumor (arrow) adjacent to the duodenum (*). **c** Axial planning CT with planned needle trajectories; tumor (red) and 5-mm safety margin (yellow) outlined. **d** Three-dimensional liver rendering showing tumor (orange), hepatic veins (purple), portal vein (cyan), and planned needle paths. **e** Planning CT orthogonal to the needle axis with tumor (red), margin (yellow), and planned needle paths (circles). **f** Fused post-placement and planning CT confirming alignment of actual (circles) and planned needle paths. **g** Planning CT in a plane parallel to the needle axis, showing planned needle positions and active tip segments (green). **h** Fused CT confirming precise needle placement. **i** Immediate post-procedure CT showing complete ablation coverage. **j** Three-month follow-up MRI confirming complete ablation with a residual parenchymal defect (arrow). CT, Computed tomography; MRI, Magnetic resonance imaging

**Fig. 2:**
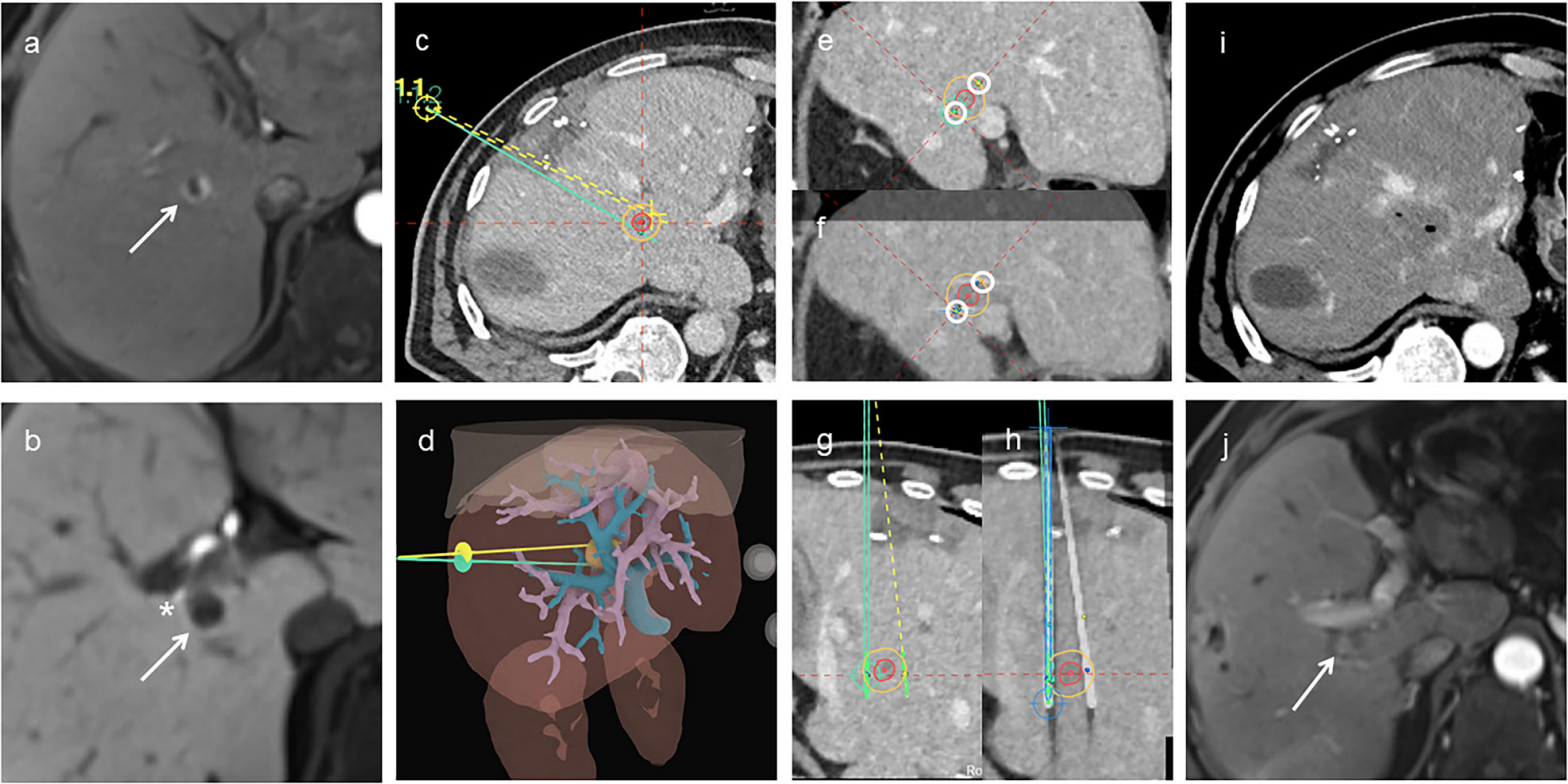
Stereotactic two-needle irreversible electroporation for neuroendocrine liver metastasis near the bile duct. **a**, **b** Axial arterial and hepatobiliary phase MRI showing peripherally enhancing tumor (arrow) near second-order bile duct (*). **c** Axial planning CT with needle trajectories; tumor (red) and 5 mm margin (yellow) outlined. **d** Three-dimensional liver rendering showing tumor (orange), hepatic veins (purple), portal vein (cyan), and planned needles. **e** Planning CT orthogonal to the needle axis showing tumor (red), margin (yellow), and planned needle paths (circles). **f** Fused CT confirming actual needle paths closely matched the plan. **g** Planning CT in a plane parallel to the needle axis, showing planned trajectories and active tips (green). Due to pleural proximity, a non-parallel orientation was selected. **h** Fused post-placement and planning CT confirming accurate needle alignment. **i** Immediate post-procedure CT showing tumor encompassed by the ablation zone. **j** Three-month MRI showing no residual tumor with parenchymal defect (arrow). CT, Computed tomography; MRI, Magnetic resonance imaging

A post-procedural dual-phase contrast-enhanced CT scan was performed to assess for immediate complications and confirm technical success, administering 100 mL of non-ionic iodinated contrast (Accupaque™ 350; iohexol 350 mg I/mL; GE Healthcare Buchler GmbH & Co. KG). Representative examples of successful stereotactic two-needle IRE procedures are shown in Figs. 1 and 2.

### Endpoints, geometrical analysis and statistical analysis

The primary endpoints were primary technique efficacy (PTE) and local tumor progression (LTP). PTE was defined as the complete eradication of the target lesion on the first follow-up imaging performed approximately 6 weeks after treatment. LTP was defined as the development of tumor recurrence at or immediately adjacent to the perimeter of the ablation zone on subsequent follow-up imaging, following an initially successful ablation [40]. Follow-up imaging was performed using multiphase MRI with a hepatocyte-specific contrast agent (Primovist, Bayer AG) at 6 weeks, 3 months, and every 3 months thereafter. Follow-up MRI was performed on a 3-Tesla system (MAGNETOM Skyra, Siemens Healthineers) using a standardized liver protocol: axial and coronal T2-weighted sequences (with/without fat suppression), T1 in-/out-of-phase, diffusion-weighted imaging with apparent diffusion coefficient maps, and a three-dimensional (3D) fat-suppressed T1-weighted gradient-echo dynamic series (bolus-tracked arterial, late arterial, and portal-venous phases), followed by a hepatobiliary phase at 20 min. MRI was used for all patients except one, in whom severe susceptibility from a gastric metal clip precluded diagnostic MRI; in that case, a dual-phase contrast-enhanced CT was performed with a non-ionic iodinated agent (Accupaque™ 350; iohexol 350 mg I/mL; GE Healthcare Buchler GmbH & Co. KG), acquiring late arterial and portal-venous phases.

Secondary endpoints included the incidence and severity of complications, which were classified and graded according to the Cardiovascular and Interventional Radiological Society of Europe‒CIRSE classification system [41].

**Fig. 3:**
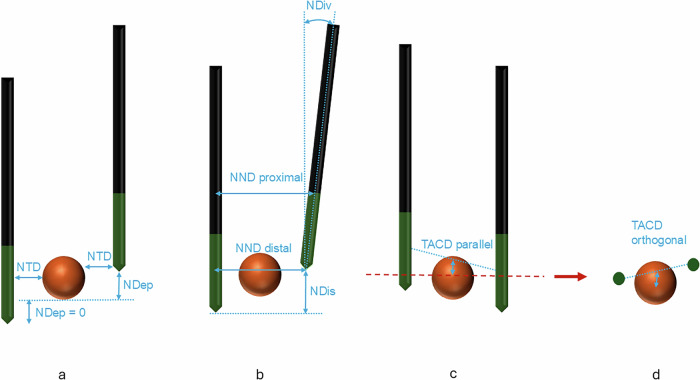
Schematic illustration of geometric parameters, adapted from Mathy et al [23]. **a** Needle-to-tumor distance (NTD) and needle depth (NDep). NDep quantifies the portion of the tumor extending beyond the needle tip. It is calculated for each needle individually and set to zero if the active tip fully covers the tumor. **b** Needle-to-needle distance (NND), measured at proximal and distal ends of the active tips. Needle divergence (NDiv) reflects the angle between needle trajectories. Needle length disparity (NDis), newly introduced, measures the vertical offset between the two tips. **c**, **d** Tumor-to-ablation center distance (TACD), measured in parallel and orthogonal planes relative to the needle axis. The overall TACD represents the spatial offset between the tumor center and the midpoint between the active needle tips (the ablation center)

Geometric parameters describing needle placement were assessed as shown in Fig. 3. All geometric parameters were adopted from the methodology described by Mathy et al [23], with minor adaptations to suit the two-needle IRE configuration. Tumor segmentation was performed on the preprocedural CT scan and fused with the post-placement CT; if necessary, a prior contrast-enhanced CT or MRI was used for image fusion. All geometric measurements were conducted using CAS-One IR software (v4.2, CAScination AG) based on reconstructed images aligned with the original ablation plan.

Furthermore, ablation parameters, including electric field strength, maximum needle temperature, and delivered current, were collected to characterize energy delivery. On immediate post-interventional contrast-enhanced CT, the ablation zone’s long and short axes were measured, and the sphericity index (SI) was defined as the short-to-long axis ratio.

Due to the small sample size and variable distribution of data, continuous variables are reported as median with interquartile range (IQR) values (25th–75th percentile). Proportions for binary outcomes (PTE, LTP, and complications) are presented as percentages with two-sided exact 95% binomial confidence intervals (CIs) (Clopper–Pearson). Normality was assessed using the Shapiro–Wilk test to support the use of non-parametric statistics. All analyses were conducted using IBM SPSS Statistics version 28.0 (IBM Corp.). A two-sided *p* < 0.050 was considered significant.

## Results

### Patient characteristics

Between December 01, 2021, and May 31, 2025, 17 patients with 18 tumors underwent stereotactic IRE with two needles. Detailed patient and tumor characteristics are summarized in Table 1.

**Table 1:** Patient demographics and tumor characteristics

Characteristic	Value
Number of patients	17
Sex	
Male	14 (82%)
Female	3 (18%)
Age, years	68 (IQR 58–77, range 41–81)
Number of tumors	18
Tumor entity	
Primary liver tumors (HCC)	12 (67%)
Secondary liver tumors (metastatic)	6 (33%)
Colorectal cancer	3 (17%)
Neuroendocrine tumor	1 (6%)
Pancreatic cancer	1 (6%)
Granulosa cell tumor	1 (6%)
Location adjacent to:	
Central bile ducts	8 (44%)
Gallbladder	6 (33%)
Stomach	2 (11%)
Small bowel	1 (6%)
Colon	1 (6%)
Tumor diameter, mm	10 (IQR 9–14, range 5–20)

Values are presented as number (percentage) or median (IQR; 25th–75th percentile); where applicable, range (min–max) is also reported

*HCC* Hepatocellular carcinoma, *IQR* Interquartile range

### PTE and LTP

PTE was obtained in 17/18 tumors (94.4%, 95% CI: 72.7‒99.9%). The single lesion without PTE underwent repeat IRE using a three-electrode configuration in a separate session to reliably cover the tumor and intended ablative margin; complete ablation was confirmed at first follow-up. The LTP case was retreated with electrochemotherapy (ECT) in a separate session because optimal needle geometry was unlikely, even with multielectrode IRE, and ECT is less alignment-dependent.

**Fig. 4:**
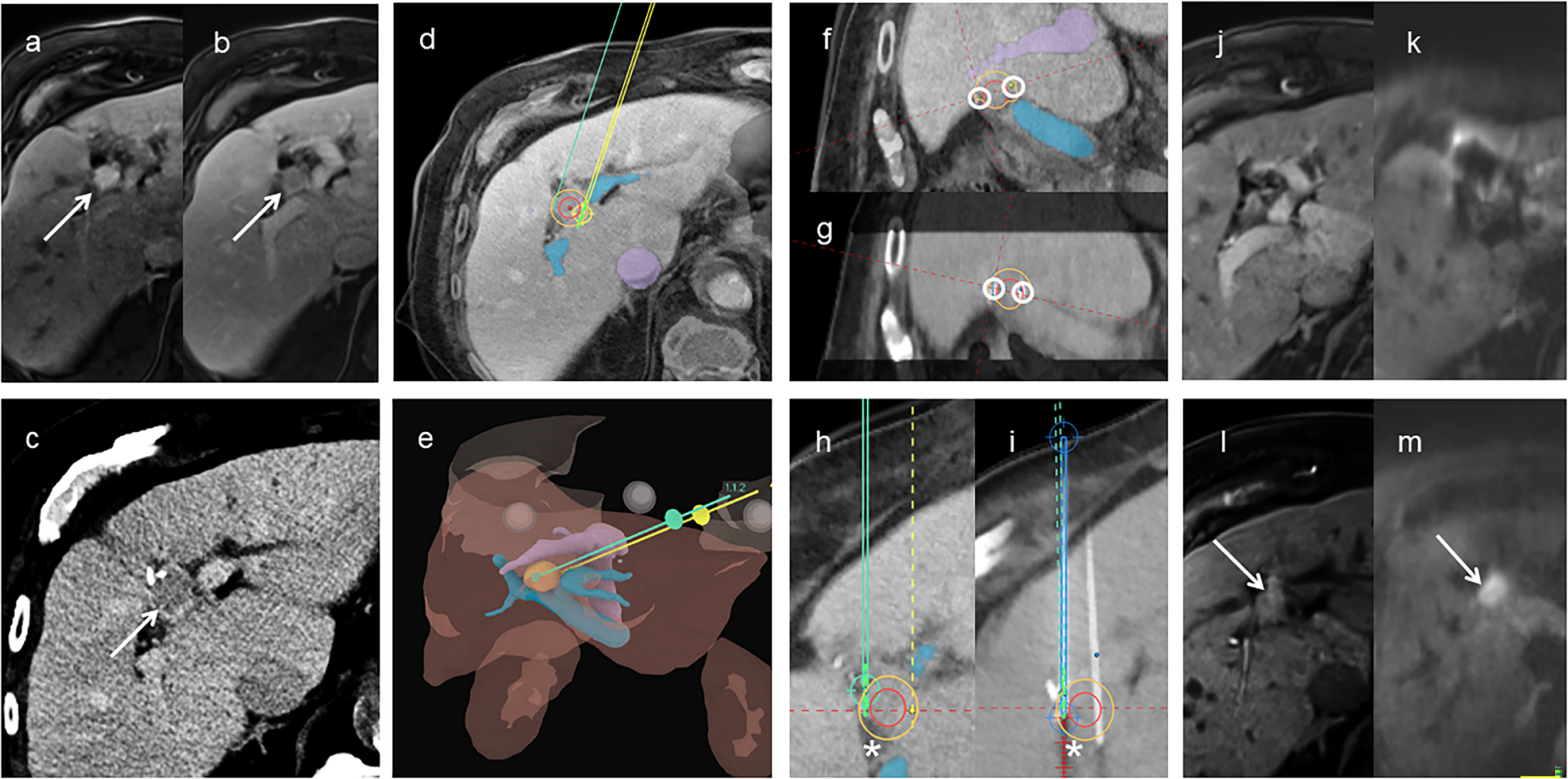
Stereotactic two-needle irreversible electroporation in a patient with hepatocellular carcinoma adjacent to the common bile duct. **a**–**c** Arterial and portal-venous phase MRI and planning CT showing the tumor (arrow). **d** Planning CT fused with MRI showing the tumor (red), 5-mm safety margin (yellow), portal vein (blue), inferior vena cava (purple), and planned needle trajectories. **e** Three-dimensional liver rendering showing the tumor (orange), hepatic veins (purple), portal vein (cyan), and planned needle paths (yellow, green). **f** Planning CT orthogonal to the needle axis showing the tumor (red), safety margin (yellow), and planned needle paths (circles). **g** Fused post-placement CT in the orthogonal plane showing minor deviation from planned needle positions. **h** Planning CT in a plane parallel to the needle axis showing planned needle trajectories, with one needle tip located near the common bile duct (*). **i** Fused CT showing significant needle length disparity (NDis = 11 mm). **j**, **k** First follow-up showing complete ablation. **l**, **m** New diffusion-restricted lesion (arrow) adjacent to the ablation zone at 17 months, consistent with local tumor progression. CT, Computed tomography; MRI, Magnetic resonance imaging

**Fig. 5:**
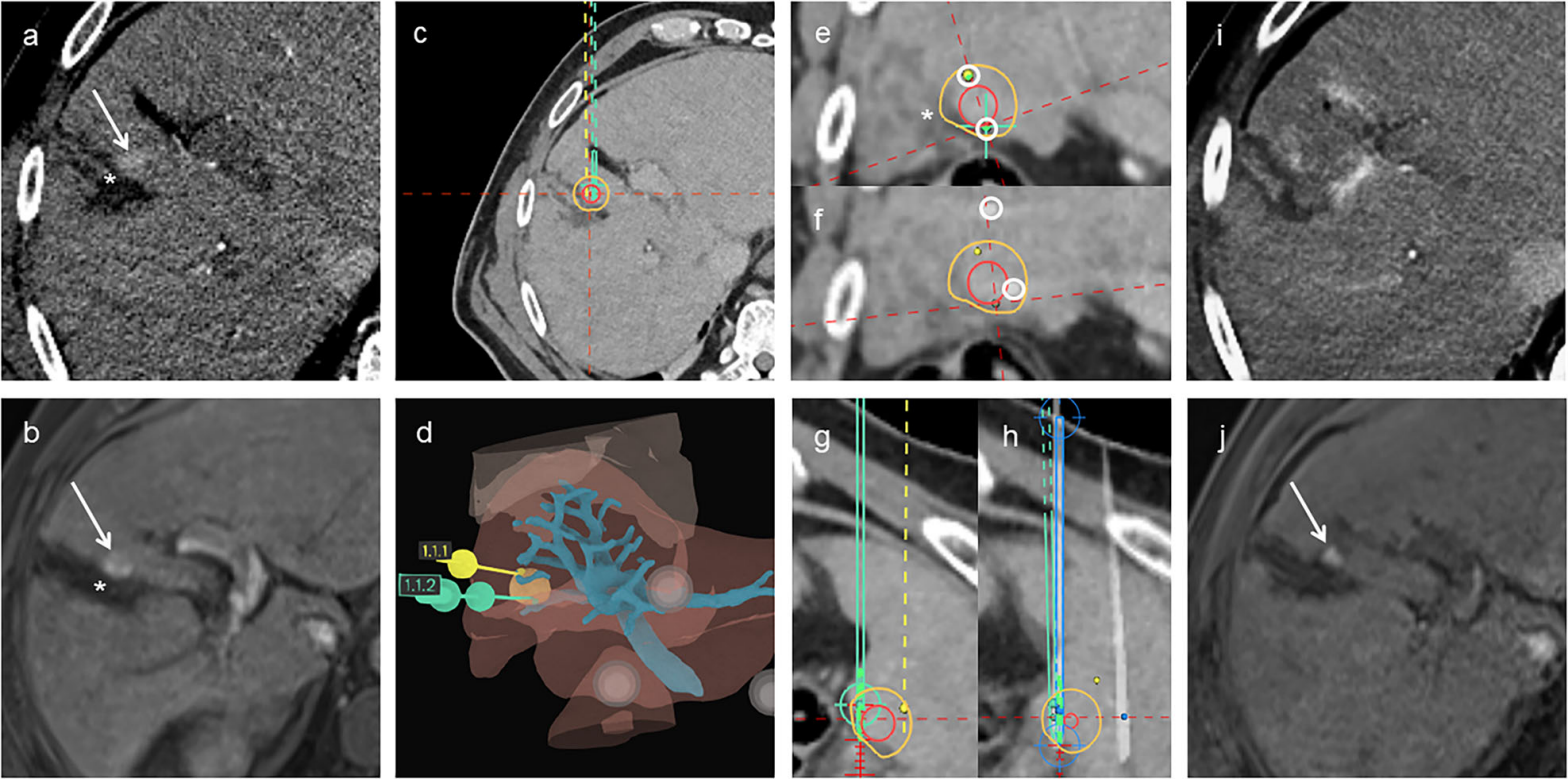
Stereotactic two-needle irreversible electroporation in a patient with HCC adjacent to the gallbladder. **a**, **b** Arterial phase planning CT and preoperative MRI showing the tumor (arrow) adjacent to the collapsed gallbladder (*). **c** Axial planning CT with planned needle trajectories; tumor (red) and 5 mm safety margin (yellow) outlined. **d** Three-dimensional liver rendering showing the tumor (orange), portal vein (cyan), and planned needle paths. **e** Planning CT orthogonal to needle axis showing the tumor (red), safety margin (yellow), and planned trajectories (circles). **f** Fused post-placement CT showing substantial misalignment from the plan and notable decentering of the ablation zone relative to the tumor (TACD = 9 mm). **g** Planning CT in a plane parallel to the needle axis showing planned needle trajectories. **h** Fused post-placement CT in the parallel plane confirming suboptimal needle alignment. **i** Immediate post-procedure CT showing ablation zone. **j** First follow-up MRI showing residual tumor (arrow). CT, Computed tomography; MRI, Magnetic resonance imaging

At a median follow-up of 12.4 months, LTP occurred in 1/18 tumors (5.6%, 95% CI: 0.1‒27.3%), occurring at 17 months. This case, shown in Fig. 4, involved a patient with an HCC adjacent to the common bile duct. Significant needle length disparity was required to avoid the bile duct, and despite initially adequate ablation, a new diffusion-restricted lesion developed adjacent to the ablation zone at 17 months, consistent with LTP. The only case without PTE is shown in Fig. 5. A patient with HCC adjacent to the gallbladder demonstrated marked needle misalignment and decentering on post-placement imaging, resulting in incomplete ablation and residual tumor on the first follow-up MRI.

### Complications

No procedure-related complications or deaths occurred during follow-up (median 12.4 months). The 30-day complication rate was 0.0% (0/17; 95% CI: 0.0‒19.5%).

### Geometric needle analysis

Geometrical parameters describing needle placement are summarized in Table 2.

**Table 2:** Geometrical needle parameters

Parameter	Value
Tumor-to-ablation center distance (TACD), mm	5 (3–8)
Needle depth (NDep), mm	0 (0–1)
Needle length disparity (NDis), mm	2 (1–5)
Needle-to-tumor distance (NTD), mm	2 (0–4)
Needle-to-needle distance distal (NND distal), mm	18 (15–19)
Needle-to-needle distance proximal (NND proximal), mm	16 (15–18)
Needle divergence (NDiv), degrees	2.0 (1.6–3.6)

All values are reported as median (IQR, 25th–75th percentile)

*NDep* Needle depth, *NDis* Needle length disparity, *NDiv* Needle divergence, *NND* Needle-to-needle distance, *NTD* Needle-to-tumor distance, *TACD* Tumor-to-ablation center distance

### Ablation and procedural data

Detailed procedural parameters are summarized in Table 3. Electric field strength, current, and maximum needle temperature data were only available for 15 of 18 tumors.

**Table 3:** Procedural parameters and ablation zone metrics

Parameter	Value
Electric field strength	
1,250 to < 1,500 V/cm	6 tumors (40%)
1,500 V/cm	4 tumors (27%)
> 1,500–2,000 V/cm	5 tumors (33%)
Current, A	25.4 (22.9–30.0)
Maximum needle temperature, °C	60 (55–68)
Number of control scans	4 (3–6)
Dose‒length product, mGy·cm	1,929 (1,772–2,483)
Intervention time, min	51 (35–90)
Ablation zone long axis, mm	34 (32–39)
Ablation zone short axis, mm	21 (19–25)
Ablation zone sphericity index	0.63 (0.57–0.70)

Continuous variables are presented as median (IQR, 25th–75th percentile), and electric field strength is reported as absolute frequency and percentage of tumors. Data on electric field strength, current, and maximum needle temperature were available for 15 of 18 tumors

## Discussion

To our knowledge, this is the first clinical study evaluating the feasibility, safety, and efficacy of a stereotactic percutaneous two-needle IRE approach for liver tumors.

Using precise stereotactic planning, a high-dose protocol, and meticulous needle positioning, our two-needle approach resulted in 94.4% PTE (17/18; 95% CI: 72.7–99.9%) and 5.6% LTP (1/18; 95% CI: 0.1–27.3%) at a median follow-up of 12.4 months. Although our preliminary outcomes fall within reported ranges for multielectrode IRE, direct comparison is unwarranted due to our small, highly selected single-center cohort. For orientation, contemporary IRE series report similar ranges for local control and safety [17, 42–45]. For context only, a meta-analysis by Yu et al, encompassing 26 studies and 807 patients, reported a pooled PTE of 86% [21]. Similarly, in a multicenter study by Frühling et al, the 12-month LTP rate was substantially higher, 60% for hepatocellular carcinoma and 75% for colorectal liver metastases [45].

No complications were observed in any of the 17 procedures. For context, Gupta et al reported an overall complication rate of 23.7%, including 6.9% major complications across 25 studies (776 patients) of multineedle IRE [19]. While direct comparisons are limited, the absence of complications in our cohort should be interpreted cautiously as preliminary evidence that stereotactic two-needle IRE can be performed safely in anatomically challenging locations.

For additional context, we benchmark our results against alternative treatments, specifically thermal ablation strategies that incorporate adjunct organ-protection maneuvers, while recognizing that differences in modality, case selection, and small sample sizes preclude direct comparison. In CT-guided RFA with hydrodissection, Liu et al reported 100% primary technical success in 15 subcapsular tumors with no hydrodissection-related complications [46]. In CT-guided MWA, Schlappa et al used angioplasty balloon interposition in 9 procedures, with 100% technical success, 88.9% local control at 11.5 months, and three major plus one minor complications not attributed to the balloon [47]. In our stereotactic two-needle IRE cohort, no separation maneuvers were required, PTE was 94.4% (17 of 18 tumors), LTP was 5.6% at 12.4 months, and no complications occurred across 17 procedures.

A key factor enabling the high efficacy observed in this study is the geometric precision of needle placement achieved through stereotactic navigation. Accurate alignment of IRE electrodes is essential for generating a uniform and effective electric field, and prior studies have shown that stereotactic systems significantly enhance placement accuracy compared to freehand techniques [27]. For example, Beyer et al directly compared stereotactic IRE with conventional CT fluoroscopy-guided IRE, demonstrating a significantly higher placement accuracy (2.2 mm *versus* 3.3 mm deviation) [29].

In our cohort, stereotactic navigation enabled high geometric fidelity in needle placement. The ablation zones were well aligned with the tumors (tumor-to-ablation center distance, TACD: 5 mm). The needles were positioned close to the tumor surface (needle-to-tumor distance, NTD: 2 mm), inserted to nearly equal depths (needle length disparity, NDis: 2 mm), kept nearly parallel (needle divergence, NDiv: 2.0°), and extended far enough to fully cover the tumor with their active tips (needle depth, NDep: 0 mm). This precise placement resulted in a median delivered current of 25.4 A, which stayed within the desired therapeutic range.

These findings are consistent with those of Mathy et al, who demonstrated that successful ablations, defined as those without residual tumor, were associated with significantly more favorable needle positioning compared to unsuccessful ablations [23]. In their study, successful cases had a TACD of 3.2 mm (*versus* 11.6 mm in unsuccessful ablations), NDiv of 3.7° (*versus* 7.0°), NTD of 1.9 mm (*versus* 4.7 mm), and NDep of 2.1 mm (*versus* 6.8 mm) [23]. The values in our cohort closely resembled those of the successful group, supporting the importance of accurate and balanced needle placement for effective ablation. The importance of needle geometry is clear from two cases: one failed to reach PTE, another showed LTP. In both cases, suboptimal geometry was evident, including either pronounced decentering or significant needle length disparity, both of which likely impaired electric field uniformity and ablation coverage.

In our series, the ablation zone geometry was moderately elongated, with a median long-axis diameter of 34 mm (IQR 32–39) and short-axis diameter of 21 mm (IQR 19–25), yielding a sphericity index of 0.63 (IQR 0.57–0.70). On immediate post-interventional CT, this corresponded to ellipsoidal ablation zones aligned with the electrode axis. By contrast, ≥ 3-electrode configurations have been reported to produce rounder, more isotropic ablations when spacing and parallelism are symmetric, consistent with modeling and technical reviews of field homogeneity, although clinical confirmation remains limited [48–50].

The two-needle IRE approach may broaden use for small hepatic tumors near critical structures by improving reproducibility, trainability, accessibility, and cost. A fixed two-electrode setup standardizes planning, placement/verification, and energy delivery, reducing inter-operator variability and easing protocol transfer. With fewer alignment decisions and placements, the technique is easier to teach and supervise and lightens the on-table workflow, potentially shortening learning curves, room time, and scheduling bottlenecks. Using two electrodes also reduces disposable use and setup time, lowering per-case costs. Together, these operational gains suggest a pragmatic path to wider adoption, pending confirmation in larger prospective studies.

In our view, the 2-needle IRE technique is best positioned within a tiered ablation strategy that complements thermal modalities. RFA/MWA remain first-line when a circumferential ≥ 5–10 mm margin is achievable at a safe distance from heat-sensitive structures. When proximity to critical structures, heat-sink effects, or a predicted inability to achieve an adequate thermal margin raises concern for injury, a two-needle IRE workflow provides a low-complexity, non-thermal alternative while preserving escalation options. It can also serve as salvage after suboptimal response or local progression following RFA/MWA, with multielectrode IRE or ECT reserved for cases needing larger or more uniform ablation zones.

This study has several limitations. It is a retrospective, single-center analysis, which introduces selection and information bias and limits external validity. The cohort is small (17 patients; 18 tumors), limiting power, widening confidence intervals, and reducing the ability to detect uncommon complications. Follow-up is short (median 12.4 months), so late LTP may be missed. There is no control group (*e.g*., multielectrode IRE or thermal ablation), which precludes causal inference and direct benchmarking of efficacy or safety. Eligibility was intentionally restricted to small lesions (≤ 2.0 cm) within ≤ 5 mm of critical structures and treated with a two-needle configuration, limiting generalizability to larger or more complex tumors. Overall, these preliminary findings should be confirmed in larger, prospective multicenter studies with longer follow-up.

In this exploratory, hypothesis-generating proof-of-concept study, stereotactic two-needle IRE appears technically feasible with a favorable safety profile for small tumors near critical structures. These preliminary findings require confirmation in larger, prospective multicenter comparative studies to establish effectiveness, safety, generalizability, and appropriate indications.

## Data Availability

The datasets are not publicly available due to patient privacy restrictions, but are available from the corresponding author on reasonable request.

## Abbreviations

CI: Confidence interval
CT: Computed tomography
ECT: Electrochemotherapy
HCC: Hepatocellular carcinoma
IQR: Interquartile range
IRE: Irreversible electroporation
LTP: Local tumor progression
MRI: Magnetic resonance imaging
MTB: Multidisciplinary tumor board
MWA: Microwave ablation
NDep: Needle depth
NDis: Needle length disparity
NDiv: Needle divergence
NND: Needle-to-needle distance
NTD: Needle-to-tumor distance
PTE: Primary technique efficacy
RFA: Radiofrequency ablation
TACD: Tumor-to-ablation center distance
